# Supplementary figures and images for: An explainable artificial intelligence approach for predicting cardiovascular outcomes using electronic health records
Source: PLOS Digit Health. 2022 Jan 18;1(1):e0000004. doi: 10.1371/journal.pdig.0000004 (PMC8975108; doi:10.1371/journal.pdig.0000004)

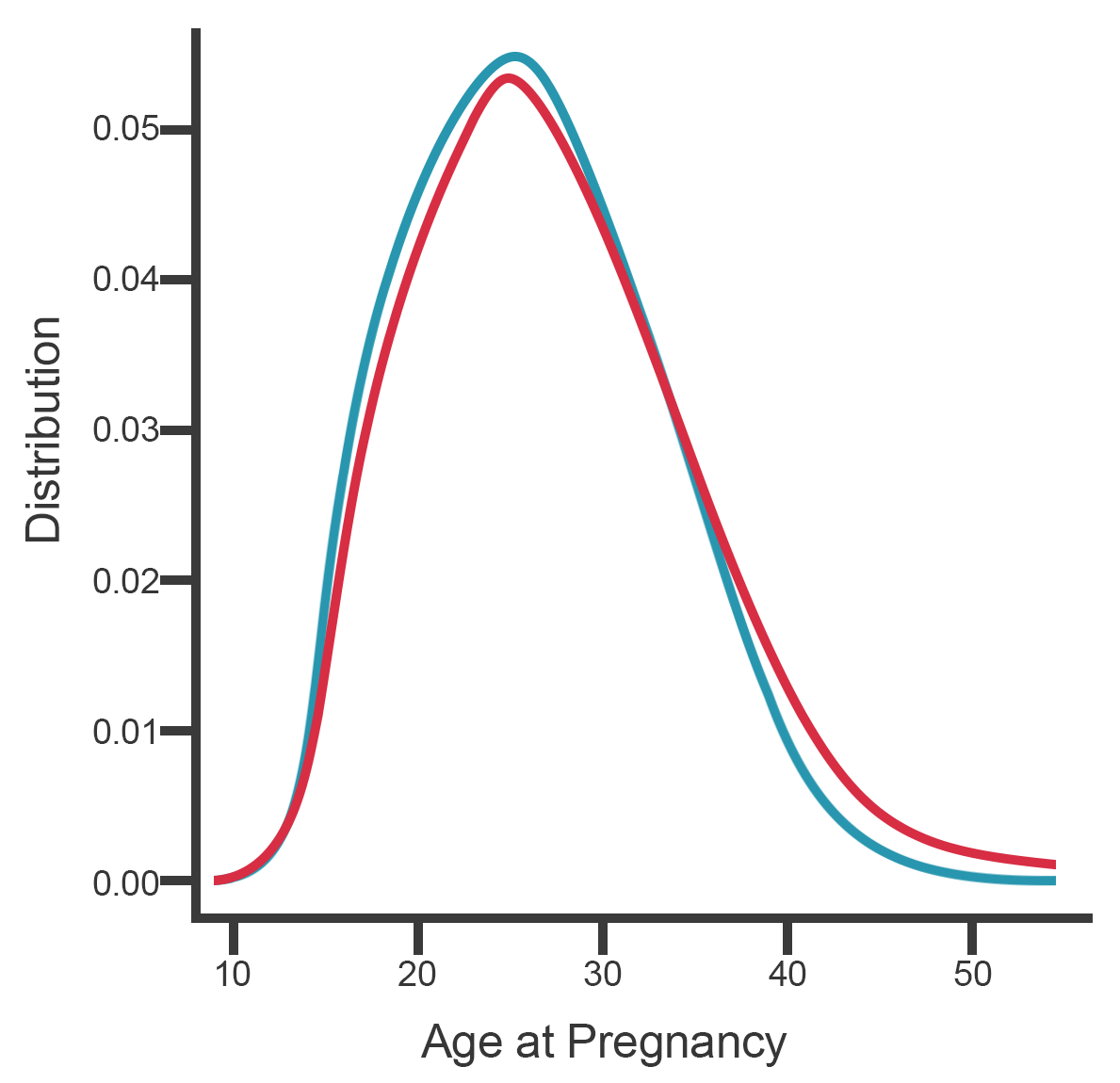

Supplement: S1 Fig — Blue line: mothers with diagnosis of hypertension complicating pregnancy (N = 11,523 mothers). Red line: mothers without diagnosis of hypertension complicating pregnancy (N = 113,491 mothers). (TIF) [file pdig.0000004.s001.tif]

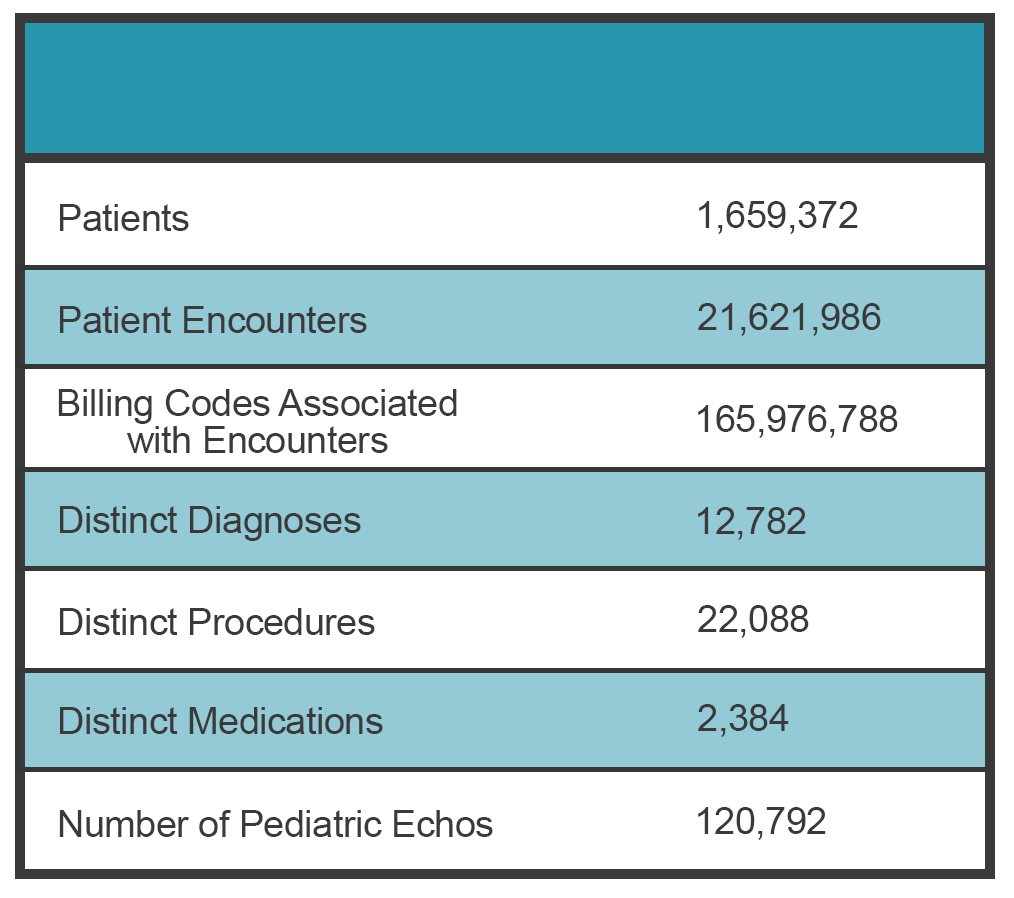

Supplement: S1 Table — (TIF) [file pdig.0000004.s002.tif]

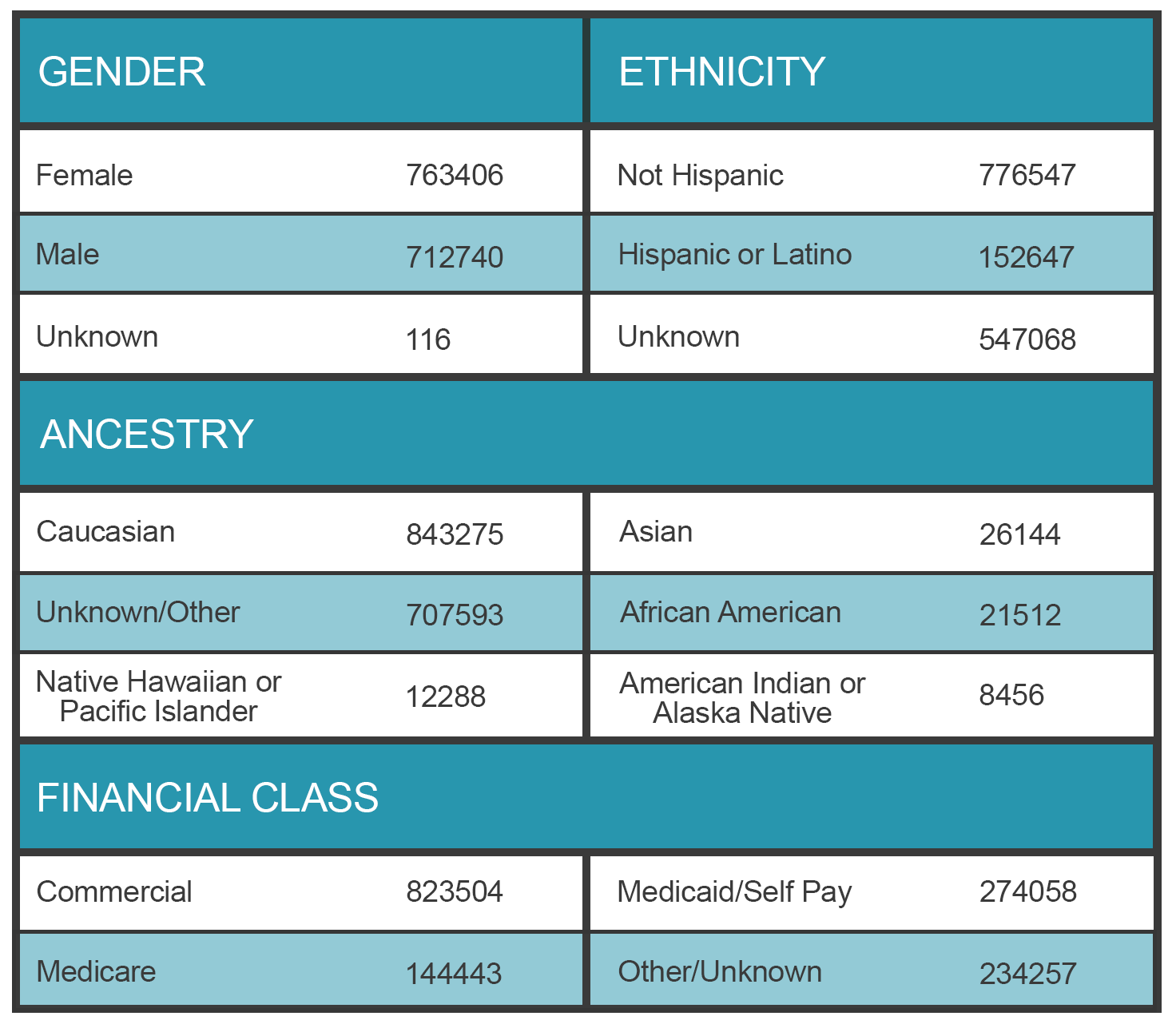

Supplement: S2 Table — (TIF) [file pdig.0000004.s003.tif]

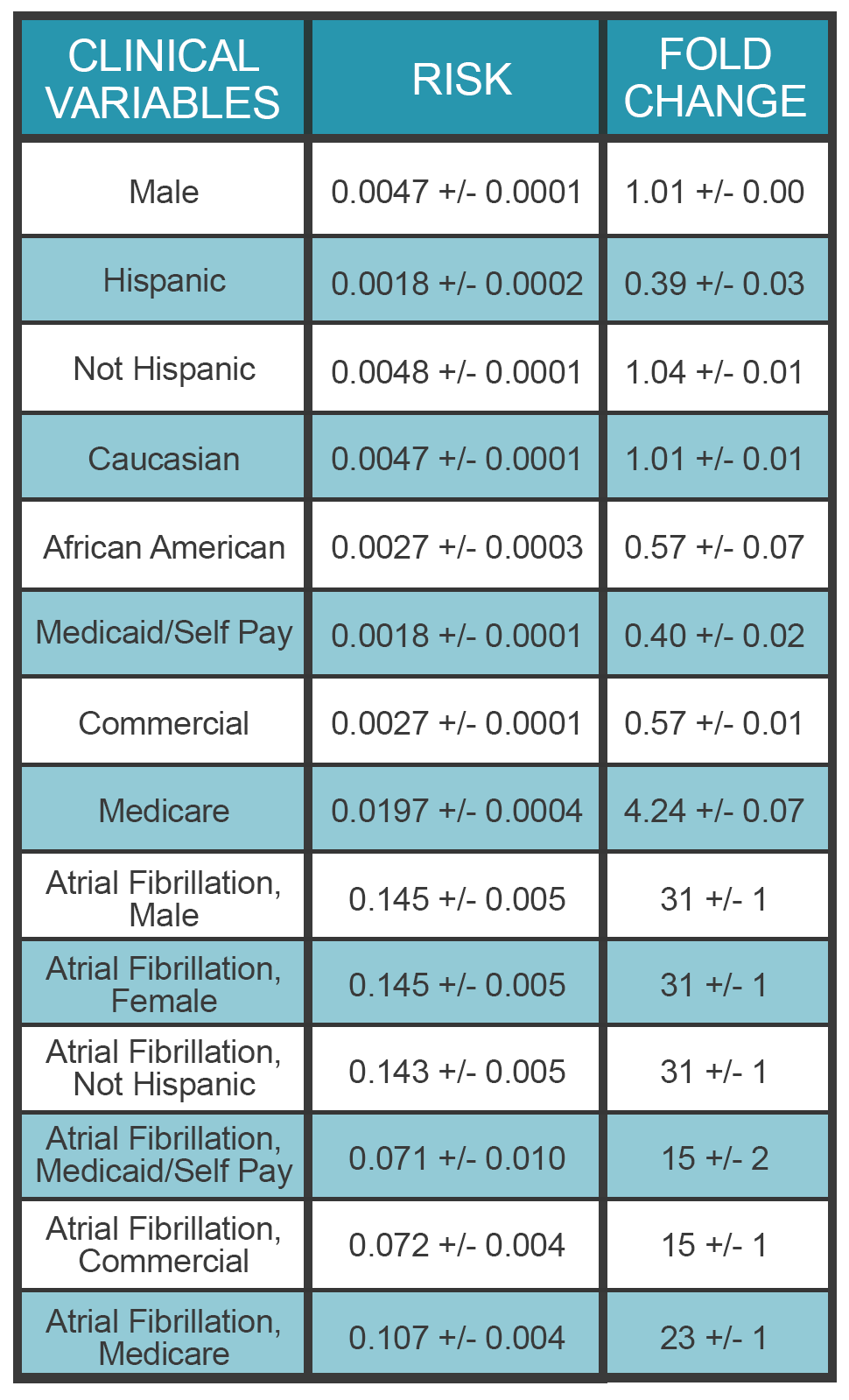

Supplement: S3 Table — Risk and fold-change risk estimates calculated from the multimorbidity network in Fig 4B main text. For detailed description of the clinical variables, please refer to S5 Table. (TIF) [file pdig.0000004.s004.tif]

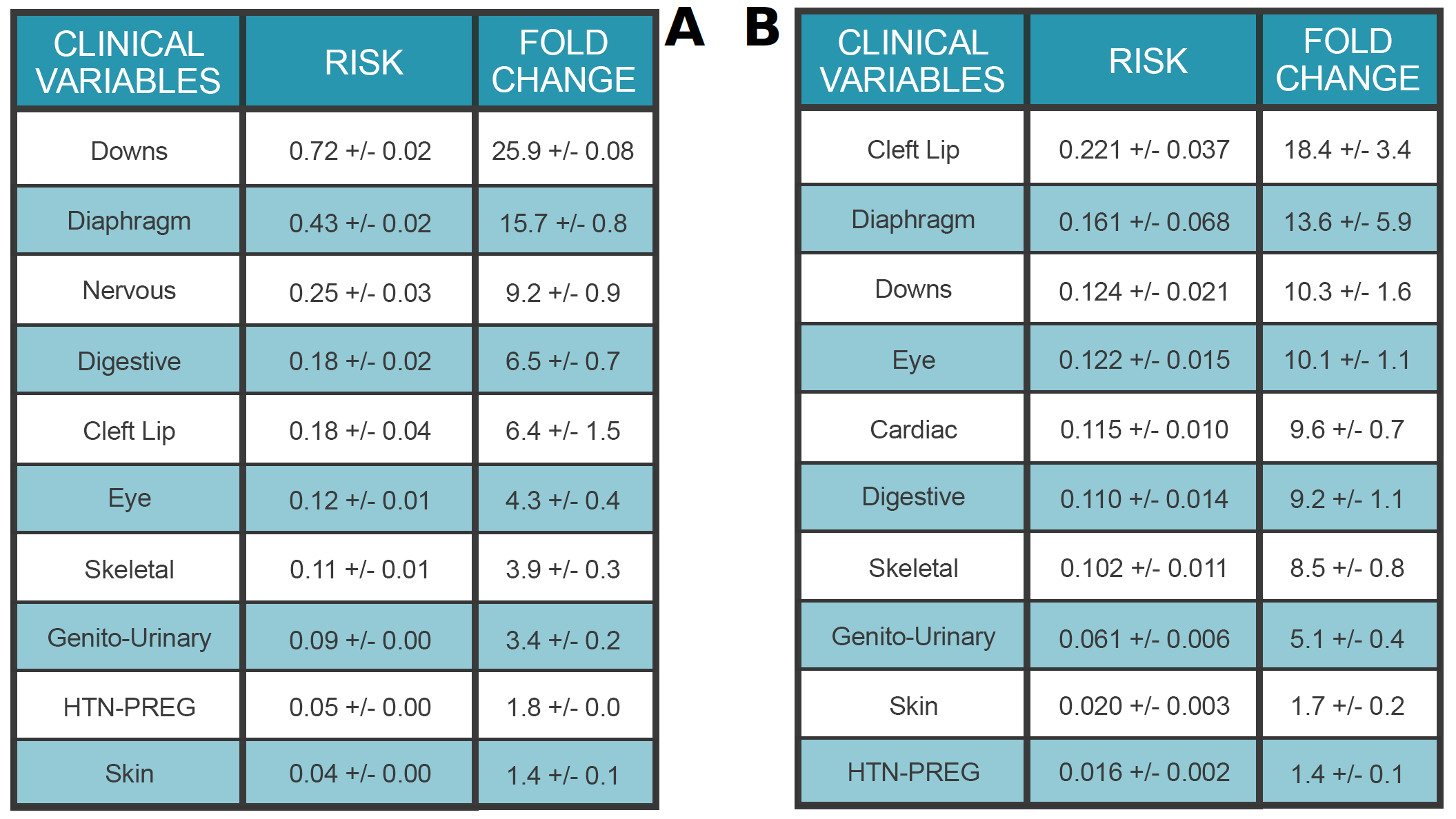

Supplement: S4 Table — Risk of cardiac (Panel A) or nervous system (Panel B) congenital anomalies given the presence of specific clinical variables. Baseline risk and fold change risk calculated using the multimorbidity network in Fig 5 of the main text. For example, a child with a known diagnosis of Down Syndrome has a 25.9-fold increased risk of a cardiac congenital anomaly over the marginal risk of cardiac anomaly. HTN-PREG, hypertension complicating pregnancy (AKA pregnancy-induced hypertension). For detailed description of the clinical variables please refer to S5 Table. (TIF) [file pdig.0000004.s005.tif]

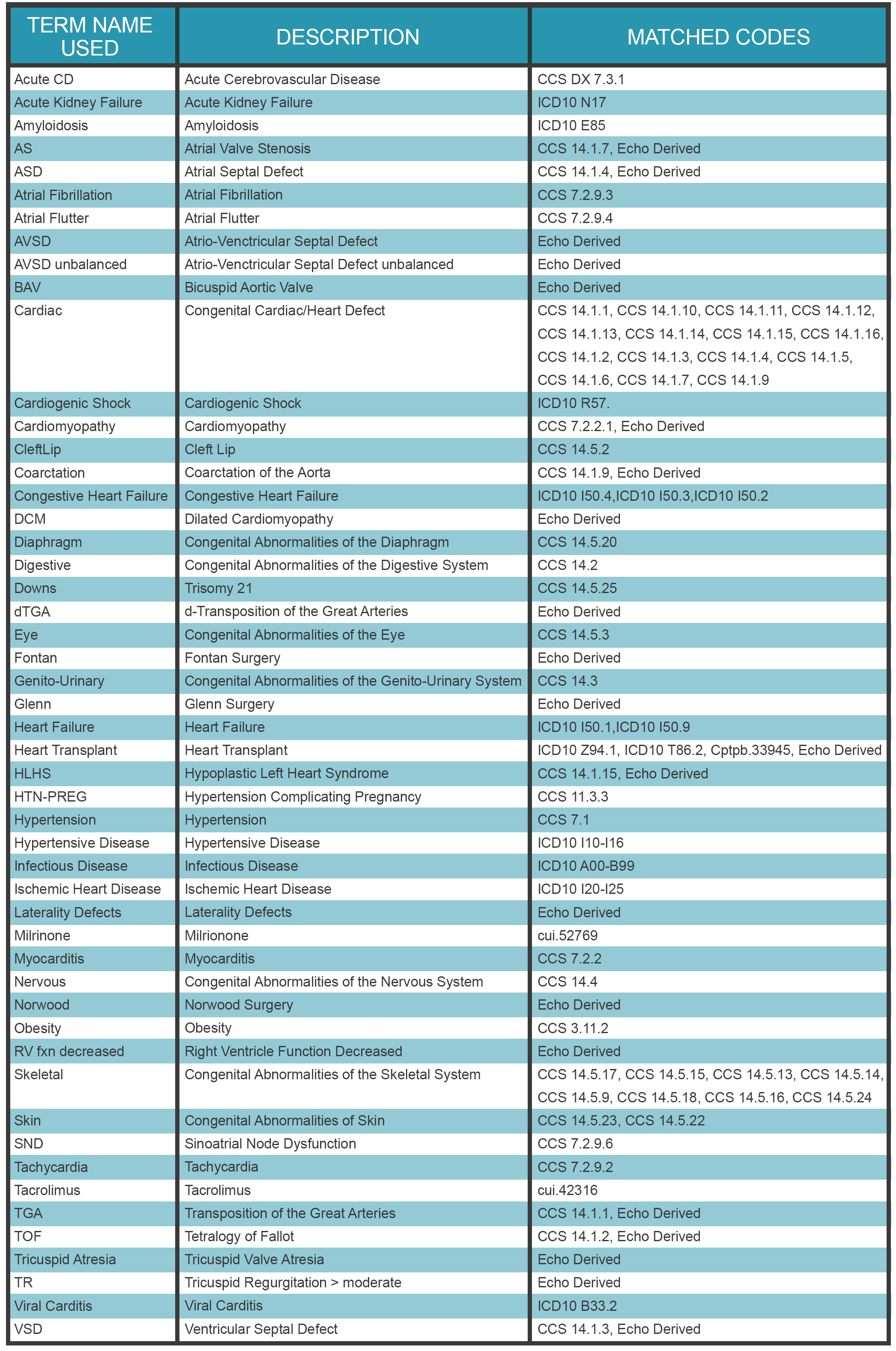

Supplement: S5 Table — (TIF) [file pdig.0000004.s006.tif]
