# Supplementary material for: An explainable artificial intelligence approach for predicting cardiovascular outcomes using electronic health records
Source: PLOS Digit Health. 2022 Jan 18;1(1):e0000004. doi: 10.1371/journal.pdig.0000004 (PMC8975108; doi:10.1371/journal.pdig.0000004)
Supplement: Attachment — Submitted filename: Response to Reviewers.docx [file pdig.0000004.s007.docx]

Response to Reviewers and Editor

*We would like to thank the reviewers and the editor for their time, and helpful feedback. Below, we have outlined our responses to each reviewer’s comments and indicated how we incorporated their remarks within the manuscript. We also used the revision process to correct some types, grammatical errors, and to smooth the text in one or two points.The result is an improved manuscript suitable for publication in PLoS Digital Health. The revision process resulted in no substantial changes to the paper’s contents, findings or conclusions.*

## Editor:

For the general readership of the journal, it would be better if you could re-organize the section headings in a typical order, e.g., introduction, methods, results, discussion and conclusions. Accordingly, that would be great if you could add a short conclusion section highlighting the limitations of the study and directions for future research.

*Thank you for your comments. We have adopted the proposed guidelines and added the suggested “Conclusion” section with highlighted limitations of the proposed approach along with directions for the future research, which was also pointed out by one of the reviewers. We have also adopted the proposed section ordering. Given the structure and focus of our work, we propose to keep the Methods and Results section in the relative order as previously submitted, but are flexible on this point too.*

Please also ensure that the link on ‘data availability’ section you provided is accessible. [<https://pbc.genetics.utah.edu/lemmon2021/bayes/bayes>]

*Thank you for capturing the link malfunction. We apologise for this inconvenience. We have reset the webpage, revised the link, and triple checked that it now directs readers to a valid webpage*

## Reviewer #1:

The authors used a comorbidity discovery method to analyse EHRs for comorbid diagnoses, procedures and medications. I believe that the paper fits well with the journal’s scope. I have a few suggestions for improving the understandability of the manuscript for a broader range of readers.

I understand that some of the authors developed the method, and the manuscript refers to the author’s previous paper where they explain the PBC. Yet, still, I would advise authors to provide more information on the method used.

I see that the authors highlighted some of the pros of using PBC, but could you please expand it? Why PBC? For instance, can’t we discover temporal relationships and quantify transition rates between comorbidities using other methods? What does PBC add to other methods?

*In response, we have expanded the description of the PBC in Methods: Patient Disease Network section, highlighting the key capabilities of PBC that make it particularly suitable for our work. We have also added a sentence explicitly directing readers to the, now published, “A Poisson binomial-based statistical testing framework for comorbidity discovery across electronic health record datasets“ article that thoroughly explains the caveats of the PBC method and provides the mentioned comparison with other methods.*

Could you please check the links provided in the manuscript?; the links do not work.

*Thank you for capturing the link malfunction. We apologise for this inconvenience. We have reset the webpage, revised the links and triple checked that they now redirect readers to a valid webpage. All hyperlinks in the article are now fully functional.*

Did your study provide some significant findings that have not been discovered yet? What does it add to the current knowledge on cardiovascular comorbidities? Would you please highlight accordingly? How can these findings be useful in practice?

*Thank you for the remark. We agree that a concise paragraph addressing this is needed in our article to better orient the reader. At the editor’s request we have added a brief conclusions section to the paper that better emphasizes the strengths, and limitations of our approach. It also (we hope) will better focus readers on some of our novel results,*  *such as the mother-child cross-generational cardiovascular multimorbidities we described.*

Could you please check Figure 4B? Some texts are overlapped.

*Thank you for capturing this display error. We have corrected the figure to match the context and we have fixed all text display issues.*

The authors might also suggest some directions for future research.

*Thank you - we have now added the future research directions in the “Conclusions” section of the article. This will certainly make the article more complete and self contained.*

## Reviewer #2:

Overall this is a thorough and interesting approach to identifying how multiple conditionally dependent variables can predict certain cardiovascular outcomes using PBC. I especially found Table 1 to drive the central point that PBC can be a powerful statistical tool in smaller sample sizes as compared with chi-squared analyses for the same variables. The other illustrations are easy to follow and support the central arguments made in the text. Overall the case is well made for the utility of PBC derived multi morbidity networks in analyzing large EHR datasets.

There is an issue with figure 4B. I believe it is a duplicated image of figure 5A. The description of Figure 4B is totally dissimilar from what is depicted in the graphic. Please consider editing figure 4B prior to publication.

*Thank you for pointing this out. We have corrected the figure duplication error and provided the correct display item to match the context. We sincerely apologize for the inconvenience.*
