# Supplementary material for: An explainable artificial intelligence approach for predicting cardiovascular outcomes using electronic health records
Source: PLOS Digit Health. Author manuscript; Available in PMC 2022 Apr 1. (PMC8975108; doi:10.1371/journal.pdig.0000004)
Supplement: Supplementary file 1 [file NIHMS1779599-supplement-Supplementary_Material.pdf]

Supplementary Display Items for “An Explainable Artificial Intelligence Approach for Predicting Cardiovascular Outcomes using Electronic Health Records”

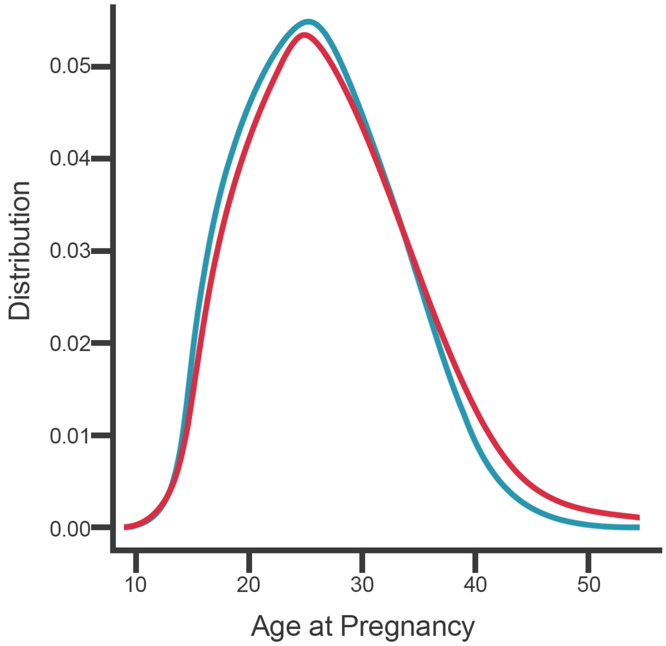

Supplemental Figure 1. Distribution density plot of mother's age at pregnancy, with and without hypertension complicating pregnancy. Blue line: mothers with diagnosis of hypertension complicating pregnancy (N=11,523 mothers). Red line: mothers without diagnosis of hypertension complicating pregnancy (N=113,491 mothers).

Supplemental Table 1. Overview of Utah Data Resource.

Patients	1,659,372
Patient Encounters	21,621,986
Billing Codes Associated with Encounters	165,976,788
Distinct Diagnoses	12,782
Distinct Procedures	22,088
Distinct Medications	2,384
Number of Pediatric Echos	120,792

Supplemental Table 2. Demographic variables and the Utah Data Resource.

GENDER		ETHNICITY	
Female	763406	Not Hispanic	776547
Male	712740	Hispanic or Latino	152647
Unknown	116	Unknown	547068
ANCESTRY			
Caucasian	843275	Asian	26144
Unknown/Other	707593	African American	21512
Native Hawaiian or Pacific Islander	12288	American Indian or Alaska Native	8456
FINANCIAL CLASS			
Commercial	823504	Medicaid/Self Pay	274058
Medicare	144443	Other/Unknown	234257

Supplemental Table 3. Multimorbidity Landscape of Sinoatrial Node Dysfunction (SND) in adults. Risk and fold-change risk estimates calculated from the multimorbidity network in **Figure 4B** main text. For detailed description of the clinical variables, please refer to supplemental table 5.

CLINICAL VARIABLES	RISK	FOLD CHANGE
Male	0.0047 +/- 0.0001	1.01 +/- 0.00
Hispanic	0.0018 +/- 0.0002	0.39 +/- 0.03
Not Hispanic	0.0048 +/- 0.0001	1.04 +/- 0.01
Caucasian	0.0047 +/- 0.0001	1.01 +/- 0.01
African American	0.0027 +/- 0.0003	0.57 +/- 0.07
Medicaid/Self Pay	0.0018 +/- 0.0001	0.40 +/- 0.02
Commercial	0.0027 +/- 0.0001	0.57 +/- 0.01
Medicare	0.0197 +/- 0.0004	4.24 +/- 0.07
Atrial Fibrillation, Male	0.145 +/- 0.005	31 +/- 1
Atrial Fibrillation, Female	0.145 +/- 0.005	31 +/- 1
Atrial Fibrillation, Not Hispanic	0.143 +/- 0.005	31 +/- 1
Atrial Fibrillation, Medicaid/Self Pay	0.071 +/- 0.010	15 +/- 2
Atrial Fibrillation, Commercial	0.072 +/- 0.004	15 +/- 1
Atrial Fibrillation, Medicare	0.107 +/- 0.004	23 +/- 1

Supplemental Table 4. Risks of Cardiac or Nervous System Congenital Anomalies as a Function of Comorbid Clinical Variables. Risk of cardiac (**Panel A**) or nervous system (**Panel B**) congenital anomalies given the presence of specific clinical variables. Baseline risk and fold change risk calculated using the multimorbidity network in Figure 5 of the main text. For example, a child with a known diagnosis of Down Syndrome has a 25.9-fold increased risk of a cardiac congenital anomaly over the marginal risk of cardiac anomaly. HTN-PREG, hypertension complicating pregnancy (AKA pregnancy-induced hypertension). For detailed description of the clinical variables please refer to supplemental table 5.

CLINICAL VARIABLES	RISK	FOLD CHANGE
Downs	0.72 +/- 0.02	25.9 +/- 0.08
Diaphragm	0.43 +/- 0.02	15.7 +/- 0.8
Nervous	0.25 +/- 0.03	9.2 +/- 0.9
Digestive	0.18 +/- 0.02	6.5 +/- 0.7
Cleft Lip	0.18 +/- 0.04	6.4 +/- 1.5
Eye	0.12 +/- 0.01	4.3 +/- 0.4
Skeletal	0.11 +/- 0.01	3.9 +/- 0.3
Genito-Urinary	0.09 +/- 0.00	3.4 +/- 0.2
HTN-PREG	0.05 +/- 0.00	1.8 +/- 0.0
Skin	0.04 +/- 0.00	1.4 +/- 0.1

A

CLINICAL VARIABLES	RISK	FOLD CHANGE
Cleft Lip	0.221 +/- 0.037	18.4 +/- 3.4
Diaphragm	0.161 +/- 0.068	13.6 +/- 5.9
Downs	0.124 +/- 0.021	10.3 +/- 1.6
Eye	0.122 +/- 0.015	10.1 +/- 1.1
Cardiac	0.115 +/- 0.010	9.6 +/- 0.7
Digestive	0.110 +/- 0.014	9.2 +/- 1.1
Skeletal	0.102 +/- 0.011	8.5 +/- 0.8
Genito-Urinary	0.061 +/- 0.006	5.1 +/- 0.4
Skin	0.020 +/- 0.003	1.7 +/- 0.2
HTN-PREG	0.016 +/- 0.002	1.4 +/- 0.1

B

Supplemental Table 5. Reference table for EHR coding.

TERM NAME USED	DESCRIPTION	MATCHED CODES
Acute CD	Acute Cerebrovascular Disease	CCS DX 7.3.1
Acute Kidney Failure	Acute Kidney Failure	ICD10 N17
Amyloidosis	Amyloidosis	ICD10 E85
AS	Atrial Valve Stenosis	CCS 14.1.7, Echo Derived
ASD	Atrial Septal Defect	CCS 14.1.4, Echo Derived
Atrial Fibrillation	Atrial Fibrillation	CCS 7.2.9.3
Atrial Flutter	Atrial Flutter	CCS 7.2.9.4
AVSD	Atrio-Venctricular Septal Defect	Echo Derived
AVSD unbalanced	Atrio-Venctricular Septal Defect unbalanced	Echo Derived
BAV	Bicuspid Aortic Valve	Echo Derived
Cardiac	Congenital Cardiac/Heart Defect	CCS 14.1.1, CCS 14.1.10, CCS 14.1.11, CCS 14.1.12, CCS 14.1.13, CCS 14.1.14, CCS 14.1.15, CCS 14.1.16, CCS 14.1.2, CCS 14.1.3, CCS 14.1.4, CCS 14.1.5, CCS 14.1.6, CCS 14.1.7, CCS 14.1.9
Cardiogenic Shock	Cardiogenic Shock	ICD10 R57.
Cardiomyopathy	Cardiomyopathy	CCS 7.2.2.1, Echo Derived
CleftLip	Cleft Lip	CCS 14.5.2
Coarctation	Coarctation of the Aorta	CCS 14.1.9, Echo Derived
Congestive Heart Failure	Congestive Heart Failure	ICD10 I50.4,ICD10 I50.3,ICD10 I50.2
DCM	Dilated Cardiomyopathy	Echo Derived
Diaphragm	Congenital Abnormalities of the Diaphragm	CCS 14.5.20
Digestive	Congenital Abnormalities of the Digestive System	CCS 14.2
Downs	Trisomy 21	CCS 14.5.25
dTGA	d-Transposition of the Great Arteries	Echo Derived
Eye	Congenital Abnormalities of the Eye	CCS 14.5.3
Fontan	Fontan Surgery	Echo Derived
Genito-Urinary	Congenital Abnormalities of the Genito-Urinary System	CCS 14.3
Glenn	Glenn Surgery	Echo Derived
Heart Failure	Heart Failure	ICD10 I50.1,ICD10 I50.9
Heart Transplant	Heart Transplant	ICD10 Z94.1, ICD10 T86.2, Cptpb.33945, Echo Derived
HLHS	Hypoplastic Left Heart Syndrome	CCS 14.1.15, Echo Derived
HTN-PREG	Hypertension Complicating Pregnancy	CCS 11.3.3
Hypertension	Hypertension	CCS 7.1
Hypertensive Disease	Hypertensive Disease	ICD10 I10-I16
Infectious Disease	Infectious Disease	ICD10 A00-B99
Ischemic Heart Disease	Ischemic Heart Disease	ICD10 I20-I25
Laterality Defects	Laterality Defects	Echo Derived
Milrinone	Milrionone	cui.52769
Myocarditis	Myocarditis	CCS 7.2.2
Nervous	Congenital Abnormalities of the Nervous System	CCS 14.4
Norwood	Norwood Surgery	Echo Derived
Obesity	Obesity	CCS 3.11.2
RV fxn decreased	Right Ventricle Function Decreased	Echo Derived
Skeletal	Congenital Abnormalities of the Skeletal System	CCS 14.5.17, CCS 14.5.15, CCS 14.5.13, CCS 14.5.14, CCS 14.5.9, CCS 14.5.18, CCS 14.5.16, CCS 14.5.24
Skin	Congenital Abnormalities of Skin	CCS 14.5.23, CCS 14.5.22
SND	Sinoatrial Node Dysfunction	CCS 7.2.9.6
Tachycardia	Tachycardia	CCS 7.2.9.2
Tacrolimus	Tacrolimus	cui.42316
TGA	Transposition of the Great Arteries	CCS 14.1.1, Echo Derived
TOF	Tetralogy of Fallot	CCS 14.1.2, Echo Derived
Tricuspid Atresia	Tricuspid Valve Atresia	Echo Derived
TR	Tricuspid Regurgitation > moderate	Echo Derived
Viral Carditis	Viral Carditis	ICD10 B33.2
VSD	Ventricular Septal Defect	CCS 14.1.3, Echo Derived
